# Children Exposed to Violence: Child Custody and its Effects on Children in Intimate Partner Violence Related Cases in Hungary

**DOI:** 10.1007/s10896-019-00066-y

**Published:** 2019-06-17

**Authors:** Júlia Galántai, Anna Sára Ligeti, Judit Wirth

**Affiliations:** 1 Linköping University, Institute for Analytical Sociology (IAS), Linköping, Sweden; 2 MTA TK Computational Social Science - Research Center for Educational and Network Studies (CSS-RECENS), Budapest, Hungary; 3 Hungarian Statistical Office, Budapest, Hungary; 4 NANE Women’s Rights Association, Budapest, Hungary

**Keywords:** Child custody, Family violence, Effects on children, Institutional violence

## Abstract

Violence might increase post-separation, and visitation can offer an opportunity to the perpetrator for maintaining power and control over the mother and child. In relationships where intimate partner violence (IPV) exists, it is hypothesized that fathers may continue their violent behaviors throughout visitation with children. The study uses mixed methods: After completing of a screening questionnaire (*n* = 593) we recruited 168 individuals from our sample with problematic child custody cases who completed an online survey. Semi-structured interviews were conducted with 30 mothers with experience of problematic child custody cases. This paper reports only the qualitative results of the research. The findings highlight how custody and visitation rights may be used as a form of custodial violence and a continuation of IPV. Problematic child custody and visitation cases were reported following separation from an abusive partner because using legal proceedings as a weapon to maintain power and control over the former partner and child. Institutions involved in custody and contact-related legal procedures do not take into consideration the violence of the abusive ex-partner as a factor when determining custody and contact arrangements, even though it may work in opposition to the child’s wellbeing. The analysis of the data shows that child custody and visitation arrangements did not reflect clear understanding of domestic violence, coercive control and the effects of these on children’s wellbeing. Fathers were reported to be able to control the everyday lives of their ex-partners and their children through lack of institutional recognition of domestic violence.

## Introduction

This study investigates child custody in Hungary, particularly in cases where visitation of a male parent is considered contrary to the child’s physical or mental well-being and safety. In some cases, violence increases post-separation, so visitation can be concerning in that it offers an opportunity to the perpetrator for maintaining power and control over the female adult victim and the child.

In recent decades in Hungary, the number of divorces has increased, with the result that – either following court-approved agreements by parents or through court decisions following litigation – children are placed at one parent’s home, with the other parent having varying visitation rights. Census data in Hungary show that in 2016, 18% of all families were single-parent families (503 thousand families). In 2016, for 87% (431 thousand families in total) of single-parent families, the mother was raising children alone, while in 13% of cases (72 thousand families) the father (Hungarian Central Statistical Office, 2016 Microcensus data). In a Hungarian representative survey, the only survey to date gathering this data in Hungary, 36% of the respondents answered that they grew up in a family where violence was present, where they feared as a child that their parents would fight, quarrel loudly, threaten each other with physical violence, or where their father had beaten their mother (Tóth 1999a; Tóth 1999b).

In 40% of adversarial divorces in Hungary, children are placed with the father. In contrast, in cases when parents can agree on their own about major issues including placement, custody and/or contact arrangements, fathers become custodial parents in only 7.7% of the cases (Grád et al. 2008).

Child custody is a complex phenomenon which is influenced by the parents of the child, family members, and also institutions that regulate the process of child-visitation and custodial rights. This process is even more complex if we analyze the effects of abusive relationships on child custody and visitation. Violence does not always end with the separation of a couple. In fact, the separation period can be the most dangerous part of an abusive relationship as the abusive person may start a battle for child custody to maintain power and control over the child and by that process over the mother as well (Hester 2000; Callaghan 2015). With no existing research on this topic in Hungary, this study represents a very first step to investigate the issue of abuse of power and control in child custody and visitation cases with a history of intimate partner violence (IPV), and its effects on its victims in the Hungarian context. This paper draws on the findings of qualitative research conducted with 30 mothers who self-identified as experiencing intimate partner custodial abuse.

Custodial or paper abuse (Miller and Smolter 2011) – the instigation of frivolous lawsuits, false reports of child abuse, and other system-related manipulations – has been recognized for some time now by practitioners and researchers as the methods perpetrators employ to continue to exert power, force contact, and financially burden their ex-partner. However, Elizabeth (2017) introduces the notion of ‘custody stalking’ with an equal focus on children, as a mechanism abusers use to control mothers following their separation. This is defined as a malevolent process involving fathers who use the custodial or legal process to overturn the historic patterns of sharing responsibilities and care of children in order to extend their control over the children and thus the mothers (Elizabeth 2017). Custody stalking is still not well recognized and is often invisible to professionals as well (Elizabeth 2017; Holt 2018; Hunter et al. 2018).

### Research Questions

International literature suggests a direct correlation between adversarial divorces and IPV, as well as pointing to the detrimental effects of IPV on post-separation child custody and visitation outcomes (Bancroft and Silverman 2002; Elizabeth 2017; Holt 2011; Holt 2018). However, no research has been carried out in Hungary to explain or contextualize these experiences. Thus, in combining the Hungarian experiences of women leaving abusive relationships and the findings of research elsewhere, our main research questions are as follows:

1. Are custody and visitation rights used as a form of custodial violence and thus a continuation of IPV in Hungary;
2. How do institutions involved in custody and contact-related legal procedures in Hungary take into consideration the violence of the abusive ex-partner as a major factor when determining custody and contact rules;

## Literature Review

Custodial violence often occurs through the exercise of irregular visitation appointments, or by means of financial exploitation through hiding joint finances or reducing support payments (Bancroft and Silverman 2002). Due to social inequalities and domestic violence, men are typically more financially secure than women, and abusers often try to buy the goodwill of the child (Emery et al. 2005).

Children exposed to violence may suffer from various behavioral and emotional problems, are more frequently referred to speech therapy (Kernic et al. 2002), more likely to be absent from school and more frequently show problematic behavior in schools, which can lead to school dropout (Byrne and Taylor 2007; Callaghan 2015). Children experiencing problematic child custody may also have impaired verbal abilities (Graham-Bermann et al. 2010) and reading skills compared with their peers (Blackburn 2008). They are also more likely to repeat classes in school (Sullivan et al. 2008). Research shows that it is not only as a direct victim of violence but as a witness to their mother’s abuse that can lead children to develop serious mental and behavioral disorders such as anxiety, difficulties establishing relationships with peers, and depression (McLaughlin et al. 2012). In some cases, the child – as a self-defense mechanism – takes up the role of the father and is later aggressive to or contemptuous about the mother, and this attitude may reoccur in the child’s future partnerships as well (Kernic et al. 2003).

Violent partners may also continue to be manipulative and maintain control over their ex-partners, thereby continuing to traumatize their children as well (Thiara and Humphreys 2017). The abusive partner also often accuses the mother of ill-treatment, alcoholism or drug use at the child guardianship office (Mullender et al. 2002), accusations which when believed can result in the child being placed in the care of the abusive parent. In this way, professional bodies such as the guardianship office, police or child welfare agencies, can be employed as weapons of institutional violence against the mother (Hester 2011; Holt 2017). In some cases, the threats or accusations that the abuser engages in to obtain custody and visitation rights becomes the primary instrument for maintaining the abuse of the mother (Holt 2018; Hunter et al. 2018).

Not all such parental behaviors fall into the category of criminal acts, but they do act to undermine the mother’s authority and parenting skills. These behaviors may also have the effect of hindering various relationships that the victims have (for example, with other family members, friends of children, co-workers, peer-parents, and teachers), detrimentally affecting the emotional well-being of the family and the development of the child (Kitzmann et al. 2003; Holt et al. 2008). Other adverse effects on the well-being of children from both direct and indirect abuse, arise from the ongoing fear that the abuser will be violent again, leading to an increase in anxiety levels (Coulton et al 2009), a higher rate of depression among children involved in problematic child custody cases, and their finding it difficult to maintain relationships as adults (Kernik et al. 2003). Using a divorce procedure to undermine the mother’s otherwise unproblematic parental behavior may itself indicate the presence of abusive behaviour on the father’s part and, as such, put his own parental skills into doubt. However, such behaviour is rarely considered to be grounds for refusing requests for custody (Humphreys et al. 2011).

Abusive fathers often blame mothers for ending the relationship, and involve children in arguments regarding the divorce which may cause additional damage to the mother-child relationship in the longer term (Radford and Hester 2006). Children that are alienated from their mothers may start communicating with their mothers with anger, distrust, or a sense of shame. They may also take on the abuser’s role by acting in a superior way, or may be ashamed to be in touch with their mother under any circumstances. This can have significant effects on the mother-child relationship (Lapierre et al. 2017) and may lead to serious personality disorders for the child (Bancroft and Silverman 2002). The abusive father might also try to manipulate the child during visitation periods to maintain control over the child and the mother. These mechanisms can be even more pronounced if the father has obtained legal custody over the child (Beeble et al. 2007).

In the last few decades, much research has been conducted about children’s exposure to domestic violence and, more recently, coercive control, which identifies potential related symptoms of post-traumatic stress disorder, a lack of social skills, and emotional and behavioral problems (Kernic et al. 2003; Överlien 2010; Holt et al. 2008; Katz 2016; Callaghan 2015). According to Bandura, who developed Social Learning Theory (Bandura 1963), children who have been exposed to domestic violence are more likely to be abusive than those who were not exposed to violence as children, a phenomenon theorized as the Intergenerational Transmission of Violence (Wallace 2005). Katz (2016) cautions however that mothers and children often provide each other with emotional support, reducing isolation and nurturing the mother-child relationship.

Children can also experience coercive control – a pattern of controlling behaviors and coercive strategies where the abuse targets the victim’s human rights: liberty, personhood, freedom and safety, and is not necessarily dependent on whether or to what extent physical violence is present – through ongoing financial abuse, monitoring and isolation, resulting in limiting their familial, social and extracurricular activities (Stark 2007; Callaghan 2015; Katz 2016). If children are enrolled in coercive behaviors, they are used as tools to exert control as direct victims of controlling and coercive acts (Hardesty et al. 2015, Callaghan 2015). It is also common that children are involved in coercive control activities by the perpetrator, including isolation, blackmailing, monitoring activities, stalking, and to legitimize violent behavior (Callaghan 2015; Stark 2007).

Research has also showed that children may not always witness acts of violence but are still aware of abusive behaviour (Devaney 2010; Överlien and Hydén 2009; Mullender et al. 2002; Överlien 2013), and should nonetheless be recognized by professionals as survivors of violence, not as passive witnesses of domestic abuse (Överlien and Hydén 2009). Kernic et al. (2003) argued that maternal distress can result in behavioral problems in children, while children who grow up in families affected by domestic violence have been shown to have a higher risk of mental health problems (Bogat et al. 2006; Meltzer et al. 2009; Callaghan et al. 2018), a higher risk of physical health difficulties (Bair-Merritt et al. 2006), and are at greater risk of encountering educational difficulties such early drop out or learning difficulties (Byrne and Taylor 2007; Callaghan et al. 2018).

When parents separate after a prior history of domestic violence, the risk to children of periods of violence and exposure to violence increase (Campbell and Thompson 2015; Lessard and Alvarez-Lizotte 2015; Broady and Gray 2018). As system theorists argue, if we include a third person in an intimate dyad, then such a relationship can be understood as a triangulation. The ordinary way of interacting in cases involving violent relationships may provoke the child to take sides or build alliances against another sibling (Callaghan 2015; Dallos and Vetere 2012). The triangulation of children during domestic violence can result in split loyalties, scapegoating, or long-term psychological distress (Callaghan 2015; Amato and Afifi 2006). To conclude, we consider that the abuse of children often occurs during IPV-related cases as a strategy for intimidating and controlling the former partner. Failing to consider this fact during the post-separation process can risk placing the child in unsafe situations (Hester 2000; Callaghan 2015).

In many cases the underlying assumption (Holt 2018; Hunter et al. 2018) of authorities seems to be that a child’s best interest is served when both parents are involved in child rearing activities. Decisions based on this assumption and granting custody or visitation to the abusive parent, even where was not involved in child care before the separation, provides perpetrators with a channel to maintain coercive control over the mother (Elizabeth 2017). In this regard, custody stalking can be a form of coercive control that humiliates and punishes women after separation, and may represent a weapon with which the mother-child relationship is weakened and attacked (Katz 2016). Despite this, it is not widely recognized by child-support authorities or family lawyers and may lead to post-separation arrangements that work against mother-child care time and the mother-child bond (Elizabeth 2017).

Custody stalking may result in the perpetrator obtaining generous visitation rights or even custody, with a corresponding involuntary loss of maternal care time following separation that may damage the psychological wellbeing of both mothers and children and have a detrimental effect on women’s mothering relationships (Elizabeth 2017). In addition, when a mother opposes the father’s award of care time in court or through legal proceedings, this can be, and often is, interpreted as alienation or hostility towards the father, and may also result in the amount of caring time for the mother being decreased (Elizabeth et al. 2010). Yet, neither of these types of malevolent attacks are widely recognized, and thus continues to hurt children and mothers.

Legal procedures and bureaucratic mechanisms of the state can be identified as a form of “secondary victimization” or “secondary abuse” using blame of the mother’s mothering style with hegemonic masculinity (Roberts et al. 2015; Heward-Belle 2017). Gender theorists claim that institutions, like families, are gendered and formal institutions reproduce what may be called the “gender regime” (Chung and Zannettino 2005, Heward-Belle 2017). Accordingly, in an invisible manner these institutions – which are meant to protect children and promote their well-being – intervene in the lives of the latter on behalf of fathers who use violence to control their partners. It is not rare that institutions minimize any violence, blame mothers for violence (Heward-Belle 2017), and in some cases threaten to grant the abuser sole custody (Saunders 2017).

We can conclude that post-separation contact involves a potentially abusive experience for children who are exposed to domestic violence (Holt et al. 2008). As research shows, one- to two-thirds of all abused women experience post-traumatic stress disorder, low self-esteem, depression and anxiety, while during legal procedures many abused mothers develop negative attitudes toward family courts and judicial systems, and feel depressed or anxious after encountering them (Elizabeth 2017).

In the following section we outline the methodology employed for the purpose of this study, before moving on to selectively present the findings, analyzing these in the context of the literature we have just reviewed in this section.

## Methods

To test our research questions, a mixed methods research design was employed, involving data collection over three distinct yet interrelated phases. Phase one involved the administration of a survey which was designed to engage both women and men who had gone through a child custody case, this involved a 10-min-long online questionnaire that was disseminated through online social media and several online magazines with a reach of hundreds of potential respondents country-wide. This survey was completed by 593 participants, who were as part of the survey completion, invited to express interest in volunteering for phase two, which involved initially filling out a 40–50 min-long second survey that focused in detail on their child custody process and experiences of IPV and also possibly participating in a semi-structured interview. The second survey, which focused specifically on those who considered their cases to have been problematic, was completed by 168 persons, 130 of whom were considered to have experienced a problematic visitation/custody case according to the criteria of our research (i.e., the partners could not agree on the child custody of their child). Among these survey respondents, 30 agreed to participate in Phase three which involved their participation in a semi-structured interview. This paper reports only on the qualitative semi-structured interviews.

Respondents in phase three of the research were female: mothers who had experienced violence during their relationships, and experienced problems with their child’s custody or contact arrangements. The interviews aimed to generate insight into IPV-related custody procedures as a whole in Hungary, but also to capture in-depth and precise information from mothers about their feelings and the effects of child custody on their children. Interviewees for phase three were purposefully selected from the list of 130 consenting participants emerging from phase two, with a view to maintaining sample variability regarding interviewees’ place of residence, age, employment status, and education level. The selected interviewees were mothers who had to have had at least one child with their abusive ex-partner, and they had to have been separated for at least two months prior to the interview. Their relationships could have been of any type (marriage, cohabitation, non-cohabitation). All participants, including the survey respondents and the interviewees, were informed that their responses would be kept strictly confidential (no sensitive data that could be connected to the respondent or their child such as address, age, school name, employer name, etc. would be released). All participants signed a consent form before the interview, indicating that they were voluntarily taking part in the research. They were also provided with the telephone numbers of civic organizations in case they wanted to seek help in the future. As the focus of the research was post-separation child custody and contact problems, in order to qualify for participation.

Before the interview started, interviewers informed all participants about the aims, and they were told that they could stop the interview if they wished to at any time (Overlien and Hydén 2009). The interviews lasted about 60–90 min on average and they were always conducted in person in a safe place with no other companion present. The interviews were semi-structured, recorded and then transcribed verbatim. No incentives were used to recruit participants. Although all the interviews were very emotional, interviewees reported that it was good to talk about their trauma and experience and felt relieved after the conversation (Vajda 2006). The interviews were paused for a break if and when needed (Overlien and Hydén 2009). An interview guide was used during the interviews and transcripts were analyzed using NVivo 10 software.

The study followed the ethical principles recommended by the Hungarian Medical Research Council’s Ethics Committee, and the proposed research process and data protection plan was officially approved by the Committee. Respondents remained anonymous.

## Findings

In the next sections we respond to our research questions by drawing on the qualitative findings, exploring if and in what way custody and visitation rights may be used as a form of custodial violence with the continuation of IPV. We also explore engagement with the institutions involved in child custody and the contact-related legal procedures in Hungary and its response to families involved in child custody and post-separation custodial abuse.

### Maintaining Intimate Partner Violence after Separation

During the interviews, some of our interviewees formulated clear explanations about the legally enforced child custody for abused partners and children and how they perceived this mechanism of abuse by the abuser. Participant mothers suggested that the acts the father commits against them are obviously not signs of care or love, but only involve a desire to maintain power over her and her child, as these next participant quotes explain:

I think he should have calmed down now. I don’t say he is holding on to me, it’s more that he had a property and he lost it. When such a man loses his property, his soul cannot find peace. (P.K.)

He was always quarreling: he has been on the phone too: ‘How are you talking to me!? Don’t dare to hang up on me!’ So, while the physical abuse stopped, the psychological abuse of the child increased. And it took me two years, and a lot of hard times, to understand that it's not that he wants to see the child, but rather that he wants to keep us in a state of fear. So, it was very hard work, and I realized that this is not love, it is absolutely abuse. (J.K.)

As the latter quote illustrates, abuse often persists after separation when there are shared children from an abusive relationship. It is also evident that this fact is not easily identified by the authorities: the abusive ex-partner’s control-maintaining mechanisms usually take covert or invisible forms such as financial deprivation, isolation, verbal humiliation, the undermining of parental authority, repeated allegations of non-cooperation, or unpredictability regarding time-keeping. It is also a common characteristic of abusers at this point to disguise such behavior as part of the ongoing coordination of placement or visitation arrangements. Besides more covert forms of abuse, access to children presents a good opportunity for the abusive parent to continue to use or commence using physical violence, which may also involve stalking and harassment. Each of these abusive methods alone, and especially their cumulative effect, has the potential to significantly influence the life of the abused ex-partner.

The overwhelming majority of the interviewees reported that children were present when the various abusive acts occurred. In some cases, the child was described as being involved as a ‘passive’ participant, witnessing their mother being abused by the father, while in many cases the child was alleged to be a direct victim of the father’s abusive behavior. Unpredictability was also a recurrent theme, mentioned as being difficult for the children to bear. Both witnessing a father’s violence and being a direct victim of physical violence are identified in this mother’s recounting of events:

When he picked a fight, I just wanted to run away. And the children were there too and saw this. He got so enraged that he grabbed the child and threw him on the bed and injured his spine. The children are afraid. (A.K.)

Fathers were also reported to behave so that their children were in a state of complete uncertainty, undermining the basic trust the latter have in their parents. In this interview, a mother reflected on the pain the father’s unpredictability had caused her children:

When the children ask him, he never tells them where they are going, or what they are going to do... When they try to ask what will happen in two weeks’ time... and he just doesn’t answer either me or the children... so they don’t like to go with him, because they are kept in uncertainty… (P.A.)

Concurring with the literature, the most frequently reported behavioral problems of children exposed to violence include anxiety, depression, learning difficulties, attention difficulties and aggression (McLaughlin et al. 2012). Anxiety and fear of the father because of past memories of violence were reported in this present study to emerge as physical symptoms or mental disorders, as the following quote suggests:

The children can’t even begin psychotherapy because their father won’t give his consent. And the Institute of Education would only admit the child after a first-instance court decision was issued. However, the middle child has already been diagnosed with depression, while the oldest boy needs full psychological treatment: He has severe self-esteem issues. Because of this he is unable to fit in, he has no friends. (H.B.)

In this study, participant mothers reported that children did not want to attend the visitation periods that they were obliged to by the authorities. Suggested reasons for this included bad memories that made them anxious about spending time with their father without the presence of the mother, but often these experiences were reported to occur during the visitation period as well. In some cases, the father was reported to not only isolate children from family members and friends, but also neglect their basic needs (like eating, learning, or keeping contact with the outside world). In such cases – as shown in the quotes below- these behaviors did not change the custody or the visitation rights of the father, even though the mother notified the authorities about what had happened to her children:

And, in the beginning, it was ‘kids, go, you must go’. Then he was on his way, and the girls said they didn’t want to go because he didn’t give them food and that it was cold. He left them, locked them in the house alone. He requested that the children be placed with him but he never applied for an extension of his visitation rights. (G.R.)

He took away their textbooks, didn’t let them study, took away their phones, and didn’t let them talk, not just with me, but with anyone. He never let them visit their friends and their friends couldn’t visit them either. He kept them in seclusion and didn’t even let them talk to the neighbors. (L.M.)

Although malicious prosecution or abusive litigation has not been extensively researched yet, it has been identified by women and by practitioners in the field for a long time (Douglas 2017). Related to both the first and second hypothesis, as our study shows how this abusive tactic may include filing civil suits in family or civil court, starting various (and numerous) procedures with a guardianship authority against the custodial parent, or pressing criminal charges based on unfounded allegations against an ex-partner (usually the custodial parent). This form of abuse was reported in most of the interviews where the mother was the custodial parent.

To further abuse the mother, abusive partners were reported to make allegations of various types (involving crimes or misdemeanors) to authorities which then kept calling the latter in for hearings and requiring them to constantly write appeals, thereby helping the father control them and manipulate authorities. One of the cases is mentioned below:

We have been in litigation for 10 years. Or rather, he is suing me, as I was always the defendant and he the applicant. He has been suing me for 10 years, sometimes at the guardianship office, sometimes in court. It is terrifying that he spends his days on this. (P.L.)

It was apparent from the interviews that unreliability in terms of complying with visitation times and dates that had been arranged was one of most obvious methods abusive ex-partners employed in order to re-establish control, and this was felt by respondents as manipulation of their and their children’s everyday lives and well-being. This form of abuse (re)creates a sense and a reality of loss of control, as one of the respondents explains:

...he doesn’t want to upset the old order... So the point is, he keeps us in a dependent position just like he did throughout our entire lives. He wants me to positively not know what will happen or when - he wants to decide about everything. (M.I.)

Another type of control and power wielded over the ex-partner involved in child custody and authorities is that the abusive father can hand in a formal accusation claiming that the mother has restricted his right to see their child, and that he could not enjoy visitation rights (Khaw et al. 2018). If a child does not want to go for a visitation because they have fear or anxiety about the father, but the father has a written, legally valid visitation right, he can hand in an accusation that will result in the mother being fined for restricting his visitation rights. In this case, the legal action can undermine the victim and involve her in long court proceedings, as the following quote illuminates:

...I received a letter saying that, because I had endangered the child [by not ensuring the exercise of contact rights], not only would the child welfare center place her in foster care, but they would also press criminal charges against me because I obstructed the visitation process. And I was completely broken. After all, I've been everywhere, I asked for help from the child welfare center, but nobody helped me, and now I will go ... [to jail] or I am deemed to have committed a crime? While the father is shouting at my daughter 'I will put your mother in jail?!’” (P.K.)

As cited in the literature, another method abusers regularly use to discredit mothers in child custody and placement cases is to allege that the woman is an unfit and irresponsible parent because of alcohol or drug use or insanity (Bancroft and Silverman 2002). Our findings also suggest that the use of this tactic tends to start before separation as a form of IPV, but it also paves the way for later litigation by perpetrators aimed at sole custody or placement. Even during the relationship, abusers manipulate their children with lies, hints, allusions and insinuations with this tactic (Bancroft and Silverman 2002). These accusations may alienate children from their mother (often the only protective relationship the child has in the family) and cause them to deprecate or despise her (Lapierre et al. 2017).

The findings from this study suggest that if these defamations are taken as valid by the authorities or court, the child can be placed with the father, totally obstructing the mother-child relationship and often leading to the total alienation of the child, as we can see from the testimonies of our interviewee:

He started a large-scale campaign against me through the children; he practically persuaded the children to mock me, to spit at me, telling them that had I ruined and devastated the entire family. He made up stories about me sleeping with strangers, forced them into fantasizing about sexual things, even going into details about what I did with men, which were not true, of course... and that I was an alcoholic and mentally ill (F.A.).

The findings of this present study highlighted how the abuser often used various types of procedures in contrary to the custodial parent, or press criminal charges based on unfounded allegations against mothers to discredit them in child custody and placement cases.

### Institutions’ and Organizations’ Roles and Contributions to Custodial Abuse

The existing literature already suggests that public agencies and institutions, such as the child guardianship authority, the police, and the courts, rarely consider that custody or visitation rights may work in opposition to the child’s welfare or safety, even if the child has directly suffered abuse, witnessed violence, or shows clear symptoms of post-traumatic stress disorder (Bancroft and Silverman 2002; Holt 2018; Khaw et al. 2018).

Our qualitative data demonstrates a pervasive lack of attention to violence on the part of authorities which handle child custody cases in any capacity. In almost all participant cases in this present study, they are reported to have failed to show any sign of recognizing the unbalanced dynamics of relationships, and the action of the perpetrator as abusive. Instead, they were reported by participants to handle such situations as if they involved mutual disagreements between partners with equal power (denying any of the effects violence can have on a victim) and equal responsibilities (implicitly or explicitly refusing to hold abusers accountable for their violent or abusive actions). A number of interviewees asserted that public agencies read the violence in the relationship as a communication problem between the couple which they together should manage. In some cases, the guardianship authority were reported to go even further, suggesting placing the child in care, claiming that both parents were too busy fighting each other and were thus unable to care for the child. This approach was experienced by participants as denying the impact of the perpetrator’s violence and holding the victim at least equally responsible for the situation. This approach was also apparent in the handling of visitation cases that were reported in this study. Visitation, and especially sleep-over contact with an abusive parent, was reported as being frequently disliked by children, who asked not to go, and in some cases mothers reported that they tried to comply with this request by not forcing their children to participate in such contact.

It was not uncommon for our respondents to report an absolute lack of recognition of the significant impact of the abuse on their children on the part of the public agencies entrusted with child protection tasks. As one mother describes her experience:

They don’t care why the children were not with him, their only point is that they weren’t with him when the father was entitled to it. And after all this they summoned me to the child welfare office and told me to sit down with the girls and tell them they had to go. They said I had to tell them this one-and-a-half days must be endured so that we could have 10 or 11 days of peace. And then the children went, but they were crying. (F.A.)

If women did not comply with such requests, they stated that they would be threatened with removal of children to foster care. Some participating women concluded that these threats were a way for the authorities to intimidate the mothers into giving up her attempts to protect the children from traumatic visitations because authorities felt overloaded by parents’ accusations and reports ‘against each other’. No visible sign that the actual merit of these accusations was being investigated was reported as apparent in most cases, thus it appears to participants that authorities assigned equal responsibility to the abusive parent who had initiated reports, and the victim who initiated reports about abusive behavior.

Another recurrent theme in the interviews as the mothers reported it was how the tactics of the abuser also worked on the employees of various agencies (guardianship office, child-welfare center) who, after experiencing accusations, harassment and threats of lawsuits, felt victimized by the father. In these situations, as mothers explained the targeted employees or the authority itself wanted to get rid of the case by passing it over to another case-handler or another authority altogether (sometimes in another city), deliberately reporting a conflict of interest as shown below from some interviewee:

He made a complaint against the social worker, so yesterday it was the fourth social worker who gave our case back. They were attacked in such a manner that they couldn’t mentally endure it. My ex-partner also said that the judge was biased, cynical, and petty.(P.K.)

I think there is an abusive person, the father; and there is an abusive office, which is the child welfare center. If I had to describe what it this like, it is the same as an abusive person. It threatens, does not pay attention to the things you do, does not understand what you say. (L.P.)

As the interviews show, wearing agency employees out with (threats of) frivolous lawsuits, complaints and retaliatory procedures against them, and the harassment of employees may lead to constant changing of caseworkers and even changes in the agencies that handle the cases. At a minimum, this was reported to facilitate the perpetrator to maintain considerable power and control over the mother’s time and money by forcing her to take extra time off and causing her expenses in travel and legal fees.

Children exposed to violence were also reported by their mothers to be violent with their peers. These behaviors and attitudes from the testimonies also show that IPV affect the school carrier, social abilities and relationships of the child:

This went so far that by third grade, my son, who had been a very good student before, became a tense child who had started to display the symptoms of a learning disorder. This is when the first report was made – a teacher notified the social worker that the child was very tense and nervous. (O.D.)

From our qualitative data we could show some insight into how authorities were reported to handle child custody cases in Hungary. Institutions were experienced as not taking into consideration the cases of IPV in relationships. The institutions were perceived by participants to be overloaded by parents’ accusations and reports ‘against each other’ and often handled the cases as ‘communication problems.’ Participants reported that the perpetrator often used abusive techniques against the authorities as well with handing in allegations against the institutions. Authorities were also reported to use threatening techniques against the mother, frightening her that her child can be taken to foster care if she did not agree on custodial cases with the father.

## Discussion and Conclusion

This research described in this paper on child custody in Hungary, particularly in cases where visitation of a male parent is in contrary to the child’ physical or mental well-being and safety. We can conclude from the findings presented above, that custody and visitation rights may be used as a form of custodial violence and thus a continuation of IPV. Our qualitative data showed that in IPV related cases abusive fathers use child custody as a form of custodial violence and thus a continuation of IPV. Our finding also confirm that the legal institutions were not experienced as recognizing the significance of IPV in child custody cases but rather promoting visitation rights for the father, resulting in the violence remaining ‘invisible’ in many cases. Our interviewees mentioned that the institutions in most cases do not realize these aims of abusers and thus provide them with the opportunity to continue behaving abusively through exercising their visitation rights, or even through granting them custody, irrespective of the harm it causes.

In the absence of any previous research in Hungary about IPV-related child custody and visitation experiences, a mixed-methods approach to obtain a fuller picture of the mechanisms, the process, and the participants’ roles in this phenomenon was employed. This paper reports only on the qualitative interviews. Acknowledging that the sample is not intended to be representative in Hungary, the findings are nonetheless important and provide a window of understanding into this issue in this jurisdiction.

The need for tools which can effectively assess the level of risk and harm potentially caused by an abusive parent should be developed in Hungary as well. The interviews we conducted suggest a lack of recognition of this need by the public agencies in Hungary. However in Hungary data on domestic violence is more or less only accessible from police and prosecution materials but unfortunately no large-scale or representative research has been conducted on intimate partner violence in the last 20 years (Tóth 2018). With this paper we would like to draw the attention of practitioners to the phenomenon of post-separation contact related intimate partner violence cases with the emphasis of focusing on the situation of children. We would also like to make an attempt to call attention of practitioners of coercive control against children in the relation of post-separation contact with the prior history of domestic violence. The need for further studies and research on coercive control against children in Hungary is also apparent.

In line with previous literature, this study indicated a correlation between pre-separation IPV and post-separation abusive practices affecting children such as custody stalking (Elizabeth 2017), paper abuse (Miller and Smolter 2011), undermining maternal authority and the mother-child relationship (Bancroft and Silverman 2002). It also highlighted previous findings the children themselves can become targets of coercive control – an expression that does not even exist in Hungarian as yet – limiting their autonomy as well as their social, housing and emotional well-being and development (Stark 2007; Callaghen 2015; Katz 2016). The study’s findings reinforce that institutions may pay less attention to these abusive behaviours than they would necessitate in order for the mothers and children to be safe (Elizabeth et al. 2010, Saunders 2017, Heward-Belle 2017). Considering that this is a completely new research area in Hungary with no previous study with this focus, further research is needed to fully verify that in Hungary, as in other countries (Bancroft and Silverman 2002; Elizabeth 2017; Holt 2018; Hunter et al. 2018), a history of IPV is a most likely predictor of malevolent custody and contact litigation. The study also indicates that in order to provide better support to children and mothers harmed by continued post-separation abuse, the very concept of coercive control and custody stalking may be imperative to introduce within the Hungarian professional and research community.

This study also points to the need for practitioners in Hungary to include the investigation of pre-separation IPV, coercive control and custody stalking into professional language and guidelines. As none of these currently form a part of the regular training of practitioners coming to contact with victims, the potential practice initiatives may include first and foremost the creation of the necessary Hungarian expressions that are currently missing from the language to enable both victims and practitioners to describe the harmful behaviors and their effect. Developing specific training courses as well as protocols and guidelines based on the findings of this, and hopefully, future Hungarian research are potential further directions. While the link between IPV and abusive use of custody and visitation rights by violent ex-partners appear to exist, research into the gendered context of this abuse is extremely rare in Hungary. Thus, for example, research into whether and how gendered expectations by authorities towards mothers and fathers affect authorities’ decisions on what constitutes abuse, control, rights and obligations could provide a basis for possible further policy and practice recommendations as according to our result the visitation rights of the father was important and foremost even though sometimes it was obviously in contrary to the child’s safety and emotional, psychological well-being.
